# Supplementary material for: Determination of Various Drying Methods’ Impact on Odour Quality of True Lavender (Lavandula angustifolia Mill.) Flowers
Source: Molecules. 2019 Aug 9;24(16):2900. doi: 10.3390/molecules24162900 (PMC6719936; doi:10.3390/molecules24162900)
Supplement: Supplementary file 1 [file molecules-24-02900-s001.pdf]

Supplementary materials table of content

Readme 2
Table 1 List of compounds of true lavender flowers EO 3
Figure 1Chromatogram (TIC) of true lavender flowers EO obtained via GC-MS analysis 5
Table 2 Sensory panel ranks 6
Figure 2 Mass spectra of unidentified compounds 7

Readme

„Supplementary materials" explanation:

Table 1 *List of compounds of true lavender flowers EO* – is a full list of all identified (within two unknown) compounds in true lavender flowers essential oil. The analysis was performed on Shimadzu GC-MS QP2020 (Kyoto, Japan). Moreover, this table is supported by ***Figure 1*** *Chromatogram (TIC) of true lavender flowers EO obtained via GC-MS analysis*, where peaks representing major EO constituents are signed.

Also, all ranks given during the sensory panel by all 39 judges are presented ***Table 2*** *Sensory panel ranks.*

Further, mass spectra for unidentified compounds are presented in ***Figure 2*** *Mass spectra of unidentified compounds* in order corresponding to **Table 1** (according to supplementary materials numeration order).

Table 1 List of compounds of true lavender flowers EO

Compound	RT	RI
α-Thujene	7.698	926
α-Pinene	7.911	933
Camphene	8.387	947
Sabinene	9.228	973
β-Pinene	9.326	976
1-Octen-3-ol	9.415	978
Octan-3-one	9.647	985
Myrcene	9.829	991
Hexanol <ethyl->	9.967	995
α-Phellandrene	10.278	1004
δ-3-Carene	10.487	1010
Hexyl acetate	10.601	1013
Terpinene <alpha->	10.708	1016
o-Cymene	10.887	1021
p-Cymene	10.979	1024
Limonene	11.134	1028
Eucalyptol	11.208	1030
cis-β-Ocimene	11.475	1038
Benzeneacetaldehyde	11.635	1042
trans-β-Ocimene	11.845	1048
γ-Terpinene	12.218	1058
cis-Sabinene hydrate	12.513	1067
cis-Linalool oxide	12.708	1072
Terpinolene	13.284	1088
Linalool	13.762	1101
1-Octen-3-ol acetate	14.148	1112
cis-*p*-Menth-2-en-1-ol	14.490	1122
3-Octanol acetate	14.589	1124
allo-Ocim-(4-trans,6-cis)-ene	14.768	1129
Camphor	15.297	1144
Isobutyrate <hexyl->	15.466	1149
Lavandulol	16.084	1166
Undeca-1,3,5-triene	16.379	1174
Terpinen-4-ol	16.505	1178
p-Cymen-8-ol	16.651	1182
Cryptone	16.800	1186
α-Terpineol	16.978	1191
Nerol	18.301	1229
Cuminaldehyde	18.701	1240
Carvone	18.837	1244
Linalyl acetate	19.279	1257
Isopulegyl acetate	20.058	1279
Bornyl acetate	20.313	1287

Lavandulyl acetate	20.471	1291
Unknown	20.556	1294
α-Terpinyl acetate	22.422	1352
cis-Geranyl acetate	22.833	1365
trans-Geranyl acetate	23.350	1382
7-*epi*-Sesquithujene	23.566	1388
*transi*Caryophyllene	24.300	1418
α-*trans*-Bergamotene	24.622	1434
β-*trans*-Farnesene	25.019	1454
Germacrene D	25.570	1481
α-*trans*-Farnesene	25.981	1502
γ-Cadinene	26.157	1513
trans-Calamenene	26.299	1522
Caryophyllene oxide	27.322	1588
1,10-*diepi*-Cubenol	27.776	1619
α-Muurolol	28.127	1645
unknown	28.812	1696
p-Camphorene	31.919	1977

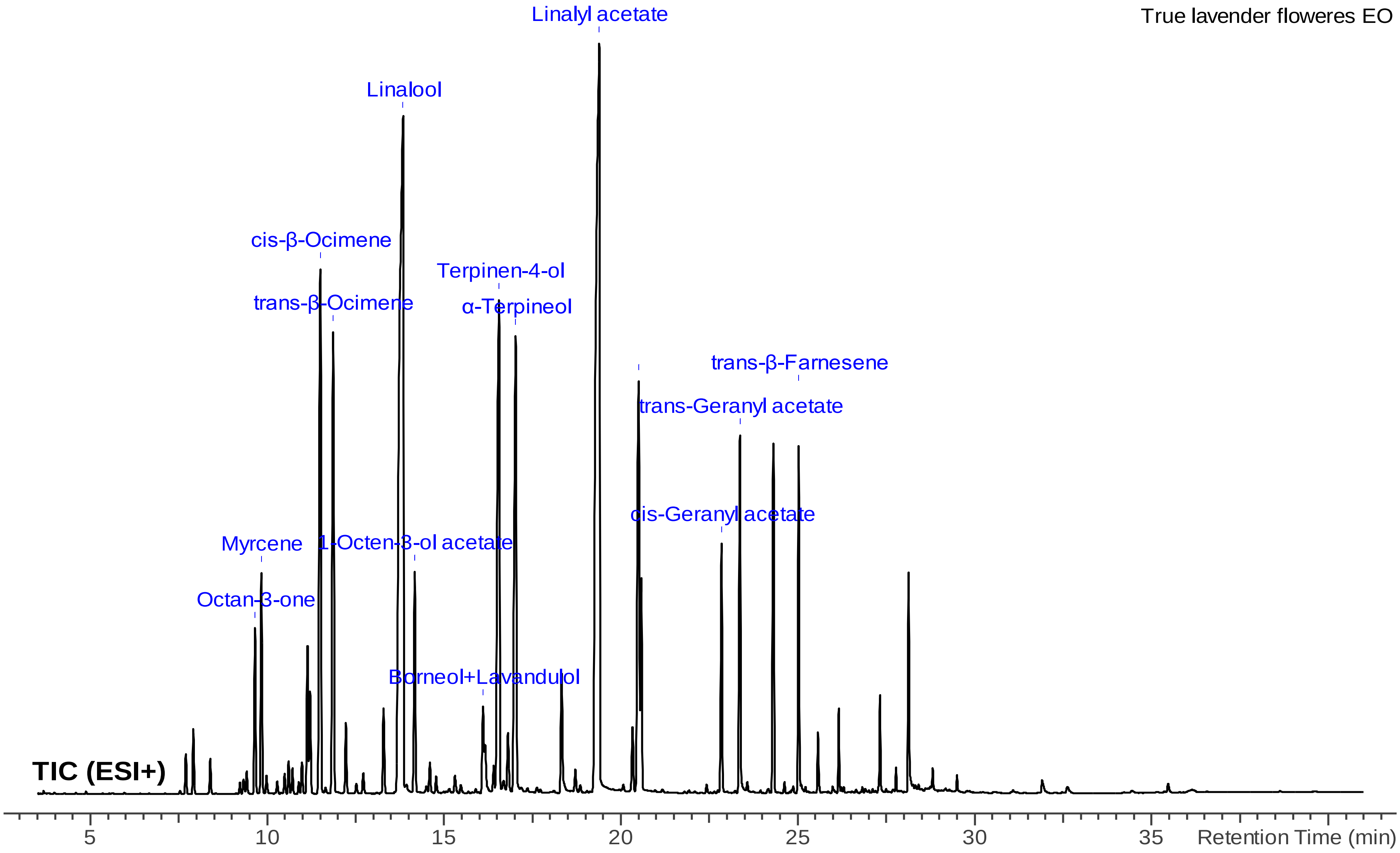

Figure 1Chromatogram (TIC) of true lavender flowers EO obtained via GC-MS analysis

Table 2 Sensory panel ranks

	Sample [code]						
Judge	CD 50 [163]	VMD 360 [672]	CD 70 [181]	VMD 240 [200]	CD 60 [95]	VMD 480 [323]	CPD60-VMFD [410]
1	1	7	3	4	5	2	6
2	1	7	2	5	4	3	6
3	1	2	3	4	5	6	7
4	1	7	2	6	4	3	5
5	2	6	1	4	5	3	7
6	4	7	2	6	5	3	1
7	3	6	1	4	5	2	7
8	6	7	3	1	4	2	5
9	6	7	4	5	3	2	1
10	6	7	1	5	3	4	2
11	3	4	5	7	1	2	6
12	4	5	1	6	2	3	7
13	4	7	1	6	3	2	5
14	1	7	3	5	6	2	4
15	4	7	2	6	3	5	1
16	7	3	1	5	6	4	2
17	3	7	1	6	2	5	4
18	5	6	2	4	7	3	1
19	2	7	6	5	1	4	3
20	1	7	3	5	4	2	6
21	4	6	1	7	3	2	5
22	3	5	1	7	4	2	6
23	3	7	2	4	5	6	1
24	5	3	1	4	6	2	7
25	5	7	6	2	3	1	4
26	3	7	1	5	2	4	6
27	3	5	1	2	4	6	7
28	7	5	4	6	2	1	3
29	7	6	1	5	2	3	4
30	3	7	1	2	5	6	4
31	7	5	4	1	2	3	6
32	7	2	5	3	1	4	6
33	7	5	4	2	1	3	6
34	7	4	6	1	3	2	5
35	7	4	5	1	3	2	6
36	7	1	5	3	2	4	6
37	7	5	6	1	3	2	4
38	6	5	7	2	1	3	4
39	7	5	4	2	1	3	6
Rank sum	**170**	**217**	**112**	**159**	**131**	**121**	**182**

	**CD50**	**CD60**	**CD70**	**VMD240**	**VMD360**	**VMD480**	**CPD60-VMFD**
**Volatiles**	1354	863	1166	786	905	840	1163
**Rank sum**	170	131	112	159	217	121	182

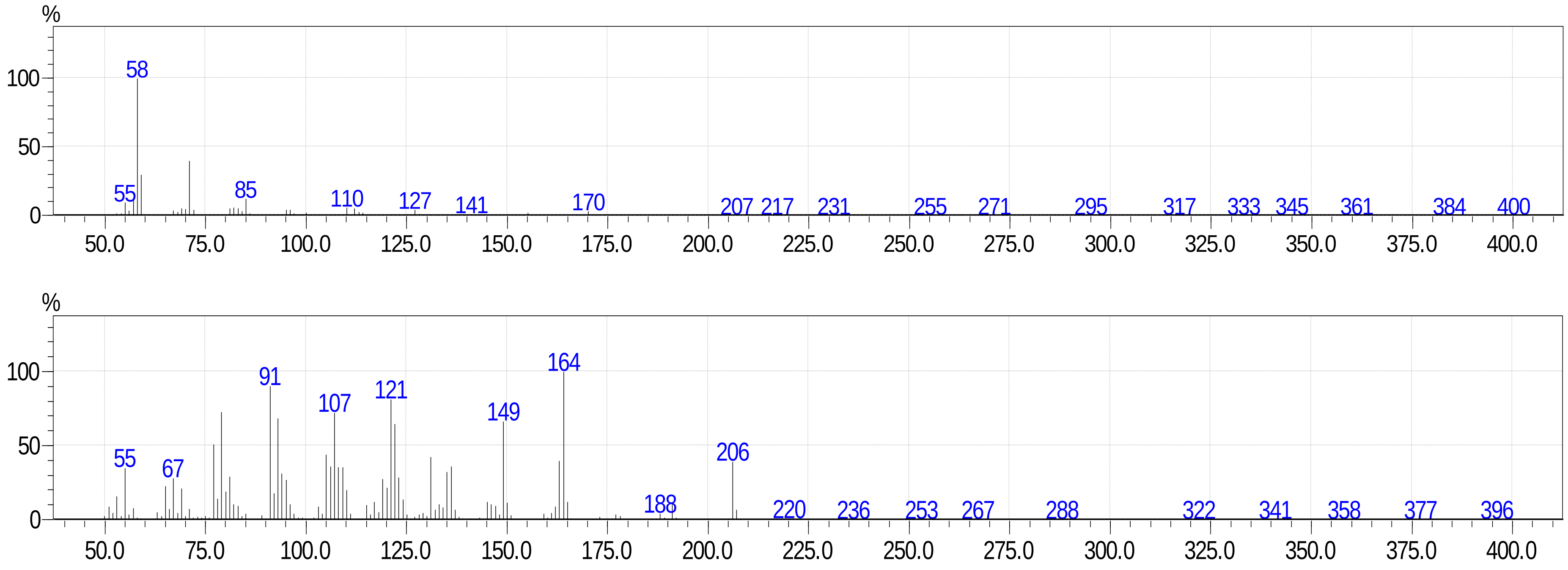

Figure 2 Mass spectra of unidentified compounds
